# New Nanovesicles from Prickly Pear Fruit Juice: A Resource with Antioxidant, Anti-Inflammatory, and Nutrigenomic Properties

**DOI:** 10.3390/cells13211756

**Published:** 2024-10-23

**Authors:** Flores Naselli, Sara Volpes, Paola Sofia Cardinale, Fabio Salvatore Palumbo, Francesco Cancilla, Francesco Lopresti, Valeria Villanova, Antonella Girgenti, Domenico Nuzzo, Fabio Caradonna, Pasquale Picone

**Affiliations:** 1 Department of Biological, Chemical and Pharmaceutical Sciences and Technologies (STEBICEF), University of Palermo, Viale delle Scienze Building 16, 90128 Palermo, Italy; sara.volpes@unipa.it (S.V.); paolasofia.cardinale@unipa.it (P.S.C.); fabiosalvatore.palumbo@unipa.it (F.S.P.); francesco.cancilla01@unipa.it (F.C.); valeria.villanova@unipa.it (V.V.); fabio.caradonna@unipa.it (F.C.); 2 Institute for Biomedical Research and Innovation, National Research Council of Italy, Via U. La Malfa, 153, 90146 Palermo, Italy; antonella.girgenti@irib.cnr.it (A.G.); domenico.nuzzo@irib.cnr.it (D.N.); pasquale.picone@irib.cnr.it (P.P.); 3 Department of Engineering, University of Palermo, Viale delle Scienze, 90128 Palermo, Italy; francesco.lopresti01@unipa.it; 4 NBFC, National Biodiversity Future Center, 90133 Palermo, Italy

**Keywords:** plant-derived nanovesicles (PDNVs), bioactive phytocompounds, antioxidant and anti-inflammatory properties, nutrigenomics, epithelial repair

## Abstract

Plant-derived nanovesicles represent a novel approach in the field of plant-derived biomaterials, offering a sustainable and biocompatible option for various biomedical applications. The unique properties of these vesicles, such as their ability to encapsulate bioactive compounds, make them suitable for therapeutic, cosmetic, and nutraceutical purposes. In this study, we have, for the first time, successfully bio-fabricated vesicles derived from Opuntia ficus-indica (FicoVes) using an efficient and cost-effective method. Characterized by a size of approximately of 114 nm and a negative zeta potential of −20.9 mV, FicoVes exhibited excellent biocompatibility and hemocompatibility, showing no reduction in the viability of human and animal cells. Our results showed that FicoVes possess significant antioxidant properties as they reduced ROS generation in TBH-stimulated cells. FicoVes displayed anti-inflammatory properties by reducing the expression of pro-inflammatory cytokines (Il 1β, TNF α) and enhancing the expression of anti-inflammatory cytokines (IL4, IL10) following an inflammatory stimulus. Furthermore, FicoVes accelerated epithelial wound closure in L929 fibroblast monolayers in a dose-dependent manner, highlighting their potential role in tissue repair. This study establishes FicoVes as a promising candidate for nutrigenomic applications, particularly in the context of inflammation-related disorders and wound healing. Further research, including in vivo studies, is essential to validate these findings and fully explore their therapeutic potential.

## 1. Introduction

Nanovesicles from various biological sources, including mammals, plants, fungi, and bacteria, have emerged as a new category of carriers. Plant-derived nanovesicles (PDNVs) are gaining increasing interest as environmentally friendly, sustainable, and biocompatible materials for the development of next-generation delivery systems [1]. Furthermore, PDNVs can themselves contain bioactive compounds of plant origin (phytocompounds), which are widely used for therapeutic, cosmetic, and nutraceutical purposes [2]. There are two types of PDNVs: extracellular vesicles (EVs), which are secreted by cells and derive from extracellular fluids (apoplastic fluid), and artificial vesicles produced during the preparation and extraction process and derived from plant destruction called EV-like vesicles.

In phyto-nanomedicine involving nanovesicles, the initial step is to prepare intact, high-yield PDNVs. Presently, the methods for obtaining PDNVs can be categorized into three main groups: the first involves the isolation and complete purification of EVs [3]; the second category encompasses the bio-fabrication of PDNVs through sonication, extrusion, homogenization, hypotonicity, air cavitation, and the use of surfactants [3]; the final category focuses on the reconstruction of PDNVs by extracting lipids from cells followed by nano-formulation processes [3]. Each method presents its advantages and disadvantages, and the advancement of new techniques, as well as the enhancement of both innovative and traditional methods, are crucial for improving the efficiency of PDNV production. EV-like vesicles are morphologically similar to the small EVs isolated from cell cultures and biofluids [4,5], and the techniques employed for their isolation and characterization are also comparable. However, due to issues such as low yield, complex isolation procedures, uncontrollable production, and challenges in scaling up for EVs, the bio-fabrication of PDNVs is becoming increasingly relevant today [3].

EV-like vesicles obtained through a bio-fabrication process from a variety of plant matrices, such as fruits, leaves, seeds, and roots, have unique properties that reflect the characteristics of the tissue of origin. After destruction of plant tissue by, e.g., homogenization, differential ultracentrifugation is the most frequently used method for the purification of EV-like vesicles. 

PDNVs exhibit high biocompatibility, and growing evidence suggests they can enter mammalian cells, facilitating plant–animal cross-kingdom gene regulation [6]. Similar to mammalian vesicles, PDNVs are small, lipid-membrane-enclosed vesicles that transport bioactive substances such as nucleic acids, vitamins, proteins, and metabolites [7]. In addition, as plants are rich in bioactive phytochemicals, research is focused on isolating plant-derived vesicles that encapsulate and transport these molecules. Most of the bioactive phytochemicals that may be present in PDNVs are secondary plant metabolites, which can be classified into three major groups: phenolic compounds (including polyphenols and flavonoids), terpenoids, and alkaloids (nitrogen-containing compounds) [8]. Many bioactive phytochemicals exhibit reduced water solubility, poor bioavailability and stability, and non-specific targeting, which significantly limit their clinical applications [9]. PDNVs can be considered as delivery systems that overcome these limitations by increasing the bioavailability of bioactive compounds through specific targeting. In addition, various bioactive molecules, chemicals, and drugs can be loaded into PDNVs to enhance the biological effect using methods such as passive incubation, freeze–thaw cycles, sonication, surfactant permeabilization, hypotonic treatment, extrusion, and electroporation [10].

Some PDNVs have been shown to have anti-cancer [6], anti-inflammatory [4,11,12], anti-aging [13], and anti-neurodegenerative [14,15] properties. Zhang et al. reported that ginger-derived PDNVs reduced inflammation in colitis-associated cancer in mice [16].

In a rat model of Alzheimer’s disease, ginger-derived PDNVs were effective in counteracting the neurodegenerative process [14]. PDNVs from grapes, broccoli, and turmeric (Curcumalonga L.) suppressed colitis in mouse models by inducing intestinal wound repair [12,16]. 

Furthermore, the use of plant-derived PDNVs as novel drug delivery systems is favored because of their intrinsic resistance to the acidic gastric environment [17], effective absorption, low cost, and sustainable production [18].

Wheatgrass-derived EVs promote wound healing by inducing the proliferation and migration of epithelial, endothelial, and dermal fibroblasts in a dose-dependent manner and stimulating the expression of type-I collagen [19]. 

The regenerative properties of grape-derived vesicles were explored in mice with induced colitis. Specifically, these vesicles were reported to penetrate the intestinal barrier, stimulate stem cell proliferation, and upregulate the expression of genes involved in pluripotency (SOX2, Oct4, Klf4) [20].

Opuntia ficus-indica, commonly known as the prickly pear cactus, is a versatile and nutritionally rich plant, highly valued in both medicinal and nutritional contexts. It thrives in arid environments and is widely cultivated in Mediterranean regions, where it is an iconic symbol of Southern Italy [21]. Prickly pear fruit has also been studied for its diuretic, hypoglycemic, analgesic, and antimicrobial effects [22]. Prickly pear extracts are renowned for their antioxidant capability and anti-inflammatory properties [23,24]. 

In this study, we have developed, for the first time, a protocol to produce vesicles derived from Opuntia ficus-indica (FicoVes). We attempted to characterize these vesicles from a physical perspective and evaluated their antioxidant and anti-inflammatory potential. Additionally, we aimed to assess their effect on wound closure capacity, exploring their epithelial repair effect. Further studies are needed to investigate the full potential and applications of vesicles produced from prickly pear fruit, but this study already establishes these vesicles as a cornerstone for their usefulness and potential applications in promoting human well-being.

## 2. Materials and Methods

### 2.1. Production and Purification of Opuntia Ficus-Indica-Derived Vesicles

The prickly pear fruits were harvested from the countryside surrounding Palermo, the capital of Sicily, and from the countryside of Ustica, a small island in the Mediterranean Sea. The juice was extracted using a manual tomato press, which was used to sieve the prickly pear fruits and separate the seeds from the pulp. A quantity of 500 mL of juice was obtained from 1 kg of fruit. The juice was then processed to obtain the vesicle. Briefly, the juices of Opuntia ficus-indica were sequentially centrifuged using an Hermle LaborTechnik GmbH-Z 300 Universal Centrifuge (220.72 v04 rotor, Hermle, Wehingen, Germany) at 900× *g* for 5 min and 2900× *g* for 5 min to remove large particles and cellular debris. At the end of these initial centrifuges, the juice was sonicated with an ultrasonic bath using FALC LBS 1 Water Bath Sonication, 5 times for 1 min. Subsequent centrifuges were carried out using a refrigerating centrifuge Awel MF 20-R (AMF 20-8 rotor) (4 °C) (Awel international, Blain, France) at 3000× *g* for 30 min, and 2 × 10,000× *g* for 30 min. The supernatant was filtered using a 0.8 μm pore filter and centrifuged at 16,500× *g* for 1 h (4 °C); then, the supernatant was centrifuged at 16,500× *g* for 3 h (4 °C). The supernatant was filtered at 0.45 μm pore filter and then it was placed at 37 °C for 1 h. It was then centrifuged using a Beckman Optima L-90K ultracentrifuge (Ti 70 rotor, Beckman Coulter, Brea, CA, USA) at 120,000× *g* for 1 h and 45 min. Then, the supernatant was removed, and the resulting pellet was carefully resuspended in 1 mL PBS. Final samples of prickly pear fruit vesicles were aliquoted and stored at −80 °C until analysis.

### 2.2. Nanoparticle Tracking Analysis (NTA) 

The sizes and concentrations of FicoVes were determined by NTA using the NanoSight^®^ Pro (Malvern Instruments, Malvern, UK). Recording and data analysis were performed using the NTA software NS XPLORER–v1.1.0.6 (Build 20000101.6). The NTA of FicoVes was performed by diluting the samples in Milli-Q water 1000, fold at 25 °C. 

### 2.3. Dynamic Light Scattering (DLS) Analysis 

FicoVes size, dimensional distribution, and Z-potential were evaluated by DLS analysis using a Zetasizer Nano ZS (Malvern Instruments, Malvern, UK). Measurements were carried out at 25 °C and with a fixed angle of 173°. Each sample was 100-fold diluted in Milli-Q water, and the particle size distribution and their Z-potential were plotted according to the results of three measurements.

### 2.4. Scanning Electronic Microscopy (SEM) 

The morphology analysis of FicoVes was undertaken through a scanning electron microscopy (SEM) model Quanta 200F (FEI, Hillsboro, OR, USA). Briefly, samples in PBS were diluted 10-fold with Milli-Q water, and 100 µL aliquots were deposited on a cellulose membrane removed from a commercial syringe filter with a pore size of 0.2 μm according to a previous study [25]. Then, samples were fixed with 2.5% glutaraldehyde and washed three times with 0.05% PBS. Thereafter, they were dehydrated progressively by dipping in 15%, 30%, 50%, 75%, and 100% ethanol v.v., three times for 3 min, and finally left to dry overnight in a fume hood. Before the analysis, the sample were attached on an aluminum stub using an adhesive carbon tape and gold-sputtered for 90 s (Sputtering Scancoat Six, Edwards Leica biosystems, Nussloch, Germany).

### 2.5. Spectrofluorimetric Analysis

The FicoVes were dispersed in PBS (pH 7.5) 1mM. UV-Vis spectra were recorded using a Spark^®^ Cyto Tecan reader (240 to 700 nm); fluorescence intensity was acquired at room temperature (25 °C) using a Spark^®^ Cyto Tecan reader (Tecan Trading AG, Switzerland) and Typhoon™ Biomolecular Imager (Cytiva Life sciences Marlborough, MA, USA). The excitation wavelengths were selected at 488–532 and 647 nm.

### 2.6. Antioxidant Properties of FicoVes

We measured the chemical antioxidant properties of FicoVes by using DPPH radical scavenging activity and FRAP ferric ion reduction capacity.

#### 2.6.1. DPPH Assay

The DPPH assay was conducted in accordance with the manufacture’s procedures of DPPH Assay Kit Antioxidant Capacity (BQC Redox Technologies- Oviedo Asturias-Spain). Briefly, the Trolox (TX) standards for the calibration curve were prepared from the 1:10 diluted standard solution according to the manufacturer’s protocol. The standard was prepared immediately prior to each assay. A quantity of 20 µL of standard or sample was mixed with 200 µL of DPPH radical solution and the absorption was measured immediately at 517 nm in a microplate reader (Glomax, Promega Milan Italy). The DPPH scavenging activity was expressed as Trolox equivalent antioxidant capacity (TEAC), using the following formula: TEAC (µM TX) = (% Inhibition of Sample -intercept/slope), in accordance with the protocol provided by the manufacturer.

#### 2.6.2. FRAP Assay

The FRAP assay was conducted in accordance with the manufacture’s procedures of the OxiSelect™ Ferric Reducing Antioxidant Power (FRAP) Assay Kit. The FRAP reagent was freshly prepared before each measurement. The standard curve was prepared immediately before carrying out the assay. The stock 10 mg/mL solution was diluted in deionized water, and this was used to prepare a series of standards according to the manufacturer. For the analysis, 100 μL FRAP reagent was mixed with 100 μL of each standard, unknown sample, or control into a 96-well plate and incubated at room temperature for 10 min. The absorbance of the solutions at 560 nm was measured by a microplate reader (Glomax, Promega Milan, Italy). Results were expressed as Trolox equivalents (eqs). Experiments were run in triplicate.

#### 2.6.3. Evaluation of ROS Generation

For acute ROS generation, 1 mM of TBH, tert-butyl hydroperoxide (Luperox^®^ TBH70X, Merck Life Science Srl, Milan, Italy) was used as a stimulus for 2 h, alone and in combination with the FicoVes (5, 25, 50 µg/mL). The control (CTR) groups received an equal volume of the fresh medium. Intracellular ROS levels were measured by using 2′ ,7′ -dichlorodihydrofluorescein diacetate (DCFH-DA). SH-SH5Y cells were plated in 96-well plates at a density of 15 × 10^3^/well, allowed to grow overnight, and incubated for 2 h with FicoVes in combination with TBH. After the incubation time, the medium was replaced with PBS buffer containing DCFH-DA (200 μM) and incubated for 15 min at 37 °C in the dark. At the end, PBS washes were performed to remove the probe and the fluorescence intensity was analyzed by a spectrofluorimeter (Glomax^®^ Explorer, Promega Milan, Italy) with an excitation of 485 nm and emission wavelength of 535 nm.

### 2.7. Cell Viability Assay

MG63, CACO2, L929, SH-SY5Y, and RAW 264.7 cells were seeded onto 96-well plates at concentrations of 24 × 10^2^, 36 × 10^2^, 18 × 10^2^, 15 × 10^2^, and 24 × 10^2^ cells/well, respectively, and treated after 24 h with increasing concentrations of FicoVes for 24 h in a humidified incubator (5% CO_2_ in air at 37 °C) [26]. Cell viability was assessed with MTT (Thiazolyl Blue Tetrazolium Bromide (MTT) BioChemica, A2231, Panreac AppliChem) assay, as previously performed [27]. The absorbance was measured at 595 nm. Three independent experiments were performed to generate the percentage of growth versus control (untreated cells) 

### 2.8. Red Blood Cells Hemolysis Test 

A quantity of 5 mL of blood from healthy human donors was processed following a previously described method [28]. After purification of red blood cells (RBCs), 200 μL of the sample was added to a 96-well plate. A range of FicoVes concentrations (1, 2.5, 5, 10, and 25 μg/mL) was then applied to the RBCs, along with 10 μL of 10% Triton X-100 as a positive control. The plate was incubated at 37 °C for 2 h. To pellet the RBCs, the plate was centrifuged at 500× *g* for 5 min. Subsequently, 100 μL of the supernatant was transferred to a clear, flat-bottom 96-well plate, and the absorbance of hemoglobin was measured at 490 nm using a plate reader (GloMax^®^ Explorer, Promega Milan, Italy). After subtracting the background, the mean absorbance was calculated.

### 2.9. RNA Extraction and Real-Time PCR 

Peripheral blood mononuclear cells (PBMCs) were grown in 12-well plates and treated with or without LPS 100 ng/mL for 4 h. Subsequently, PBMCs were treated with 5–25 µg/mL of FicoVes, for another 24 h. RNA was extracted using a commercially available kit (Qiagen GmbH, Hilden, Germany) according to the manufacturer’s instructions. Total RNA was reverse-transcribed to cDNA using a QuantiTect Reversion Transcription Kit (Qiagen GmbH, Hilden, Germany). RT-QPCR was performed in 96-well plates using the Step-One Plus Real-Time PCR system (Applied Biosystem, Carlsbad, CA, USA). For quantitative SYBR^®^Green real-time PCR, a Quantinova SYBR^®^Green PCR Kit and Quantitect^®^ Primer Assay were used and were purchased from Qiagen. Amplification was performed with 25 ng of total cDNA and β-ACTIN expression was monitored for a quantitative internal control. Relative changes in gene expression between control and treated samples were determined with the ΔΔCt method. Final values were expressed as fold change.

### 2.10. ELISA Test

Pro-inflammatory cytokine levels (IL-1β) and the anti-inflammatory cytokine IL-10 were quantified using specific ELISA kits (RAB0273, Millipore, Darmstadt, Germany;) (SEA056Hu, Cloud-Clone Corp., Wuhan, China). The assays were performed using culture supernatants of PBMCs treated with LPS 100 ng/mL for 4 h and/or 5-25 µg/mL of FicoVes for 24 h. All procedures followed the manufacturer’s instructions. The absorbance was finally measured in a microplate reader (Glomax, Promega Milan, Italy) at 450 nm.

### 2.11. Scratch Assay 

L929 cells were seeded onto 24-well plates at concentrations of 9 × 10^4^ cells/well and, once sub-confluent, the monolayer was scraped three times in parallel with a 200 uL pipette tip and a perpendicular line was drawn with a permanent marker, as described by Luparello et al. (2022). Cells were exposed to two concentrations of FicoVes (5 and 25 μg/mL), while the culture medium was replaced with plain medium for the control wells; cells were incubated at 37 °C in a 5% CO_2_ atmosphere. The scratches were photographed in the same place at various points under a phase-contrast microscope at time intervals up to 24 h from the start of the assay (T.0, after 3 h, after 6 h, after 22 h). The size of the scratch after repair compared with the initial scratch area at each time point was measured as described by [29] through ImageJ software (v. 1.53e, National Institutes of Health, Bethesda, MD, USA).

### 2.12. Statistical Analysis

Statistical analysis was conducted using Prism 10.3.1 for Windows (GraphPad Software Inc., San Diego, CA, USA). We employed one-way analysis of variance (ANOVA) followed by Tukey’s multiple comparison. A significance level of *p* < 0.05 was used to determine statistically significant differences. Data are presented as means ± standard deviation (SD).

## 3. Results

### 3.1. Production and Characterization of Opuntia Ficus-Indica-Derived Vesicles

**Figure 1:**
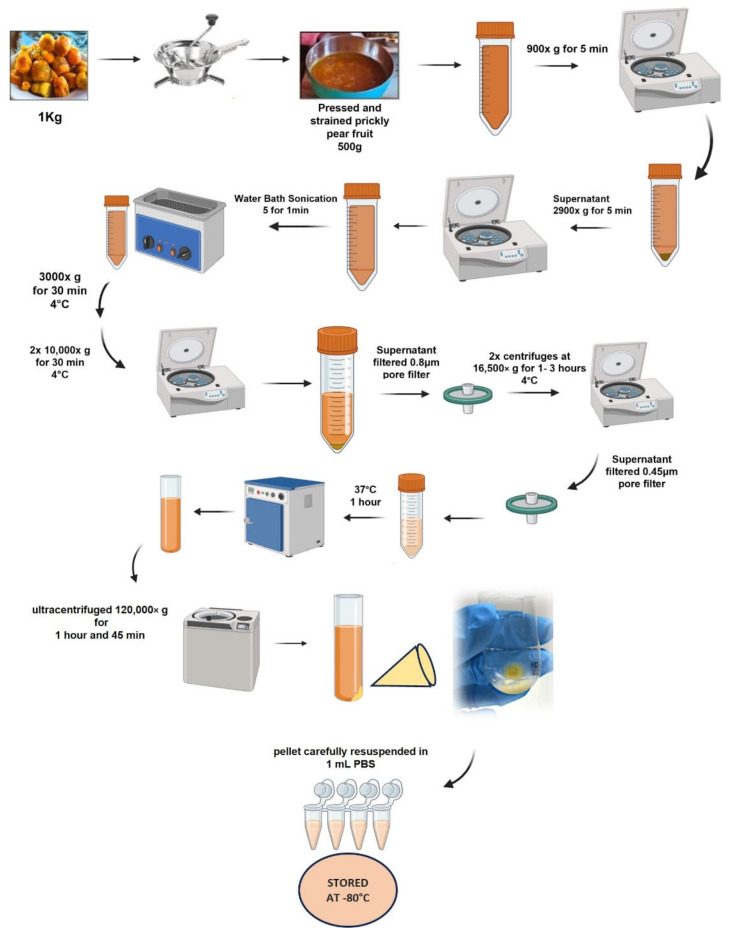
Schematic of the workflow used for the isolation and purification of vesicles from Opuntia ficus-indica. Differential centrifugation was used to prepare samples enriched in vesicles. Further separation and purification of isolated samples were performed by ultracentrifugation (UC).

**Figure 2:**
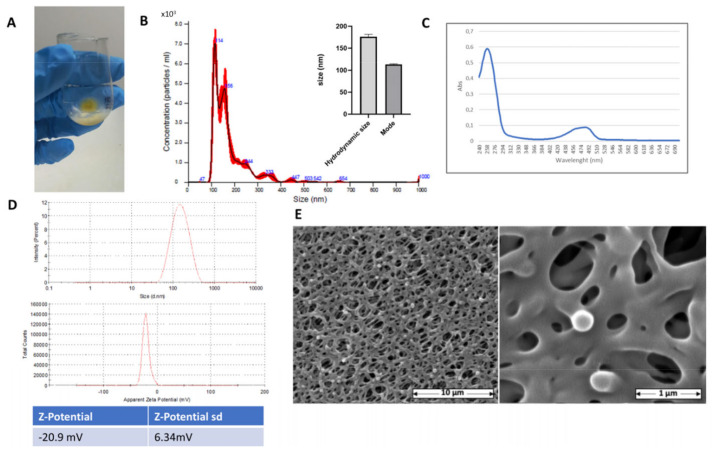
Isolation and characterization of FicoVes. (**A**) Yellow pellet obtained after ultracentrifugation at 120,000× *g* for 90 min at 4 °C. (**B**) Size distribution of FicoVes obtained through NTA. (**C**) Absorbance spectrum measure of FicoVes, spectrum range of 240–700 nm. (**D**) DLS analysis to investigate FicoVes size distribution. (**E**) Representative image of SEM analysis of FicoVes.

The FicoVes were produced by sequential centrifugations, sonication, and filtrations after being crushed and juiced from fresh prickly pear fruits, as shown in Figure 1. The techniques of nanoparticle tracking analysis (NTA) and dynamic light scattering (DLS) were used to determine the concentration and size of vesicles from Opuntia ficus-indica. These methods are commonly used in studies of vesicles of both animal and plant origins [20,30,31]. The concentration of vesicles isolated from 500 mL of Opuntia ficus-indica juice by sequential ultracentrifugation (FicoVes) (Figure 2A) was assessed using nanoparticle tracking analysis (NTA). The NTA size distribution indicated that there were two predominant populations of 114 nm and 156 nm. The total yield was 4.94 × 10^12^ ± 1.43 × 10^11^ particles/mL (Figure 2B). DLS analysis was also performed to confirm the size of FicoVes and whether they carried a charge. The data showed that the particles had a Z average size of 161.7 nm (Figure 2D) with a PDI of 0.205 and that they carry a zeta potential of −20.9 ± 6.34 mV. Since Z-potential is a significant characteristic of the stability of nanoparticles, this result indicated that the negative charge of FicoVes can facilitate the stability of nanoparticles. Scanning electron microscopy, shown in Figure 2E, highlights that the surface of the cellulose filter is covered by homogeneously distributed and round-shaped elements. The inset, at higher magnification, shows that the sizes of the spheres were consistent with the NTA analysis, thus indicating that the elements are FicoVes fixed on the cellulose membrane. It is worth noting that despite the biological material originating from different sources (as we collected fruits from various regions and compared the characteristics of the vesicles), the resulting product is consistent.

As many biologically active compounds in plant vesicles have distinct absorption and/or fluorescence spectra, we aimed to evaluate the absorption spectrum of FicoVes. Spectrophotometric absorption measurements of FicoVes over the extensive range from 240 to 700 nm show the maximum absorbance at 258 nm (Figure 2C). This absorption peak is characteristic of several phytocompounds, including flavonoids, phenolic acids, and other aromatic compounds, which are known to absorb in the UV range, particularly between 250 and 280 nm [32]. The literature suggests that bioactive compounds found in plant extracellular vesicles (PDEVs), such as polyphenols and flavonoids, often exhibit absorption peaks in this region, suggesting the presence of these complexes in our sample [33]. We also observed another absorption peak at 485 nm, which is typical of carotenoids according to the literature [33].

**Figure 3:**
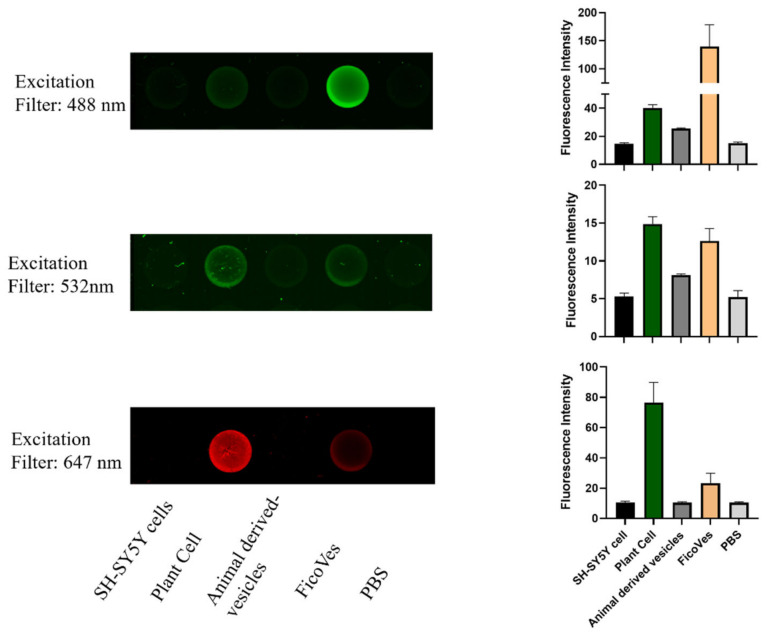
Fluorescence detected by Typhoon and the histogram related to the fluorescence intensity.

To further confirm the presence of these phytocomplexes, we performed fluorescence analysis by exciting the samples at specific wavelengths. Using appropriate excitation and emission filters, we successfully detected fluorescence, supporting the conclusion that these bioactive molecules are indeed present (Figure 3). In addition, we performed a comparative analysis of similar phytocomplexes in animal cells, animal-derived vesicles [34], and plant cells using fluorescence spectroscopy for quantification. As shown in Figure 3, when excited at 488 nm, we observed higher fluorescence in FicoVes compared to that emitted by plant cells. This suggests an enrichment of fluorescent compounds within the FicoVes. The absence of the same fluorescence in animal cells and animal-derived vesicles suggests that these phytocomplexes are specific to plant cells. Conversely, other excitation filters used consistently highlight the specificity of phytocomplexes typical of plant cells, but these are present in smaller quantities in the FicoVes compared to plant cells.

### 3.2. Antioxidant Properties of FicoVes

**Figure 4:**
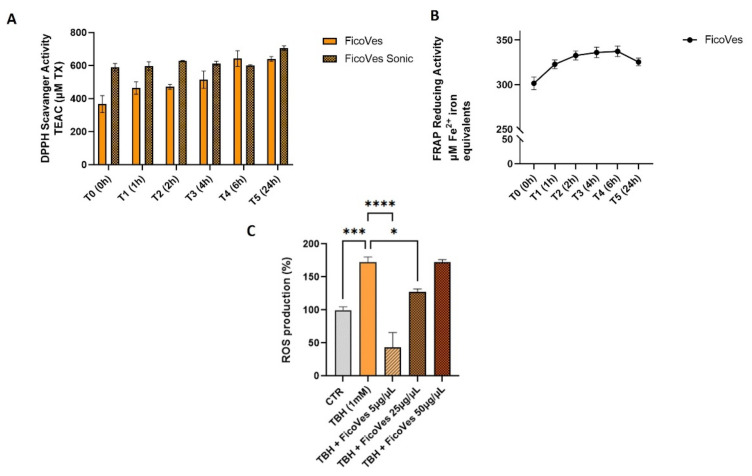
Antioxidant properties of FicoVes by (**A**) free radical scavenging (DPPH); (**B**) ferric reducing antioxidant power (FRAP). (**C**) Histogram of DCFH-DA in acute oxidative stress in SH-SY5Y cells induced by 1 mM of TBH for 2 h alone or in combination with FicoVes (5–25–50 µg/mL). Statistical analysis performed with one-way analysis of variance (ANOVA) and Tukey’s multiple comparisons test. Differences were considered significant at * *p* < 0.05, *** *p* < 0.001, **** *p* < 0.0001.

The antioxidant properties of FicoVes were studied through free radical scavenging (DPPH) and ferric reducing antioxidant power (FRAP) assays. Initially, we determined the radical scavenging capacity of the compounds through the DPPH assay. Along with the intact vesicles, we proceeded to sonicate them in order to disaggregate the vesicles to possibly release their contents into the solvent, thereby allowing us to better evaluate their antioxidant properties, since we hypothesized that the antioxidant activity was due to molecules contained within the vesicles. Figure 4A shows that FicoVes (25 µg/mL) have a TEAC of approximately 400. The vesicles that were sonicated showed a TEAC value of 600, as expected, higher than that of the FicoVes possibly due to the increased levels of antioxidant substances following ultrasonic disruption, leading to an enhancement in their antioxidant capacity. Moreover, it is interesting to note that the antioxidant capacity of FicoVes increases over time, unlike the antioxidant power of sonicated FicoVes, which remains stable. This leads us to hypothesize that the components responsible for the antioxidant activity are not immediately available, as if they were on the surface membrane, but become available gradually over time.

The reducing properties were determined through a blue complex formation using the FRAP assay. Figure 4B shows the ability of FicoVes to reduce Fe^3+^ to Fe^2+^. Additionally, the kinetics of the reducing capacity remain stable for up to 6 h, with only a slight decrease starting after 24 h. The in vitro antioxidant activity was evaluated on neuronal cells, which are known for their ability to counteract minor variations in ROS levels [35] (Figure 4C). In the experiments, SH-SY5Y cells were treated with FicoVes for 2 h in combination with 1 mM TBH to induce intense oxidative stress. The fluorescence intensity, which is proportional to ROS production, increased following treatment with TBH, used as a positive control. However, fluorometric analysis revealed a significant decrease in TBH-induced ROS generation in the presence of FicoVes. Indeed, the simultaneous treatment with FicoVes reduced ROS production to below the levels observed in the untreated control (FicoVes 5 μg/mL) and also compared to the positive control (FicoVes 25 μg/mL). In contrast, treatment with TBH and FicoVes at 50 μg/mL did not reduce ROS production compared to the positive control. These results underscore the potent antioxidant properties of FicoVes in mitigating oxidative stress in the SH-SY5Y cell system. We hypothesize that at higher concentrations, the vesicles might aggregate, forming larger structures and losing their antioxidant effect, since the 50 µg/mL dose shows the same effect as TBH alone. This hypothesis was investigated using NTA analysis, where we examined the size of the vesicles at concentrations of 5 µg/mL and 50 µg/mL. Indeed, at higher concentrations, these vesicles form larger structures, exhibiting more heterogeneous and larger populations compared to the approximately 200 nm population observed at the 5 µg/mL concentration (data showed in Supplementary File, Figure S1).

### 3.3. Effect of FicoVes on Cell Viability

**Figure 5:**
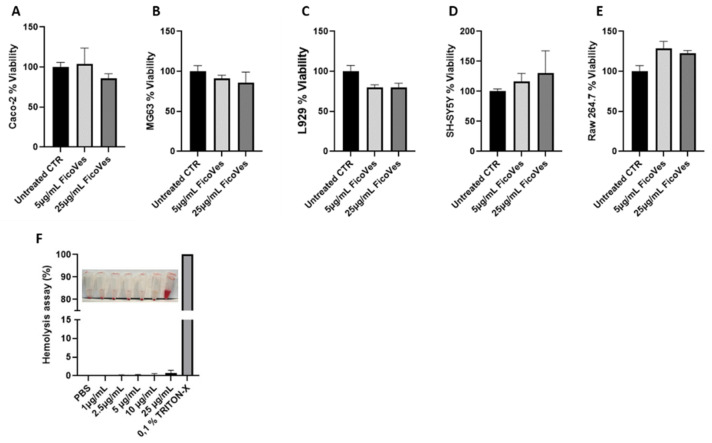
Effects of FicoVes on cell viability following treatment with FicoVes (5–25 µg/mL) for 24 h. (**A**) Viability of Caco-2 epithelial intestinal cells, (**B**) osteosarcoma cells MG63, (**C**) L929 murine dermal fibroblasts, (**D**) SH-SY5Y neuroblastoma cells, (**E**) RAW 264.7 murine macrophages. (**F**) Hemolysis assay RBC treated with 1, 2.5, 5, 10, and 25 µg/mL of FicoVes. Data are presented as means +/− SEM.

To assess the cytocompatibility of FicoVes, we treated several cell lines with FicoVes at 5 µg/mL and 25 µg/mL for 24 h (Figure 5A–E). FicoVes did not significantly alter the viability of any of the cells tested at any concentration after 24 h. In fact, viability levels were above 70% in all lines tested, as suggested by the guidelines [36].

The hemolysis test was performed to assess the hemocompatibility of FicoVes. Red blood cells (RBCs) were incubated with FicoVes for 2 h. The results indicate that no significant hemolysis was observed after incubation of the RBCs with 1–25 μg/mL FicoVes at 37 °C for 2 h (Figure 5F). These findings confirm that FicoVes is hemocompatible across all tested concentrations.

### 3.4. Effect of FicoVes on the Expression of Genes Involved in the Inflammatory Pathway

**Figure 6:**
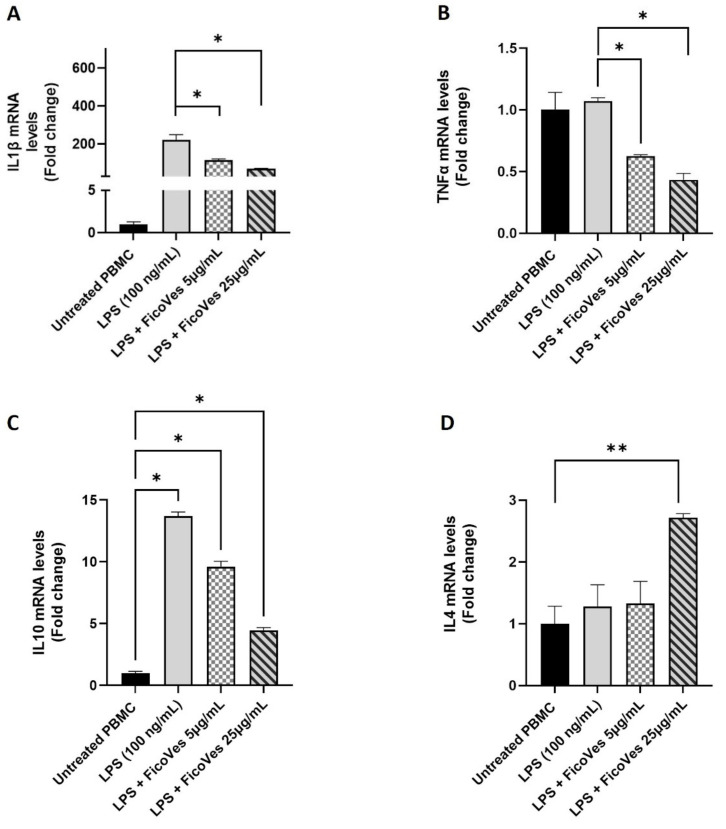
Effect of FicoVes on expression of pro-inflammatory cytokines in LPS-stimulated PBMCs. (**A**) IL-1β, (**B**) TNFα, (**C**) IL10, and (**D**) IL4 mRNA expression in FicoVes-treated cells followed by induction of inflammatory response by LPS (100 ng/mL) treatment. Data in the histograms are presented as the mean of three different experiments. Statistical significance was determined by comparison with the control group not treated with LPS/FicoVes. Statistical analysis performed with one-way analysis of variance (ANOVA) and Tukey’s multiple comparisons test. Differences were considered significant at * *p* < 0.05, ** *p* < 0.01.

Plant-derived vesicles possess remarkable biological functions, particularly in the fight against inflammatory diseases and tumors, due to their unique content of lipids and miRNAs and phytocompounds [14,37]. Given these characteristics, we investigated the anti-inflammatory properties of FicoVes by assessing the expression levels of pro- and anti-inflammatory cytokines. To induce inflammation, PBMCs were treated with LPS for 4 h. After treatment with LPS (100 ng/mL), PBMCs were treated or not with different concentrations of FicoVes (5, 25 μg/mL) for 24 h, after which cytokine gene expression was analyzed (Figure 6).

The treatment with FicoVes at concentrations of 5 and 25 μg/mL reduced the expression of IL1β and TNFα compared to the positive control (cells treated with LPS). Regarding the anti-inflammatory cytokines IL10, treatment with FicoVes at concentrations of 5 and 25 μg/mL increased IL10 expression. However, treatment with LPS alone also increased IL10 expression, as it is well-known that LPS can stimulate the release of IL10 through the AKT pathway [38]. To confirm that the increase in IL-10 expression was due solely to the vesicles and not the presence of LPS, we treated the cells with vesicles only, without LPS treatment. The results show that FicoVes at 5 and 25 µg/mL significantly increase IL-10 expression (data shown in Supplementary File). Furthermore, the expression of the anti-inflammatory cytokine IL4 was significantly increased in response to the combined treatment with LPS and FicoVes only at 25 µg/mL. We also evaluated the protein expression of the cytokines whose gene expression showed the most significant variation following treatment with LPS and FicoVes at different concentrations (IL1β and IL10). The results of the protein expression confirmed the gene expression data for the analyzed cytokines (Supplementary File, Figure S3).

Overall, these results suggest that in the presence of an inflammatory event, FicoVes reduce the expression of pro-inflammatory cytokines in a dose-dependent manner, while also appearing to increase the expression of anti-inflammatory cytokines.

### 3.5. Effect of FicoVes in the Epithelial Repair Process

**Figure 7:**
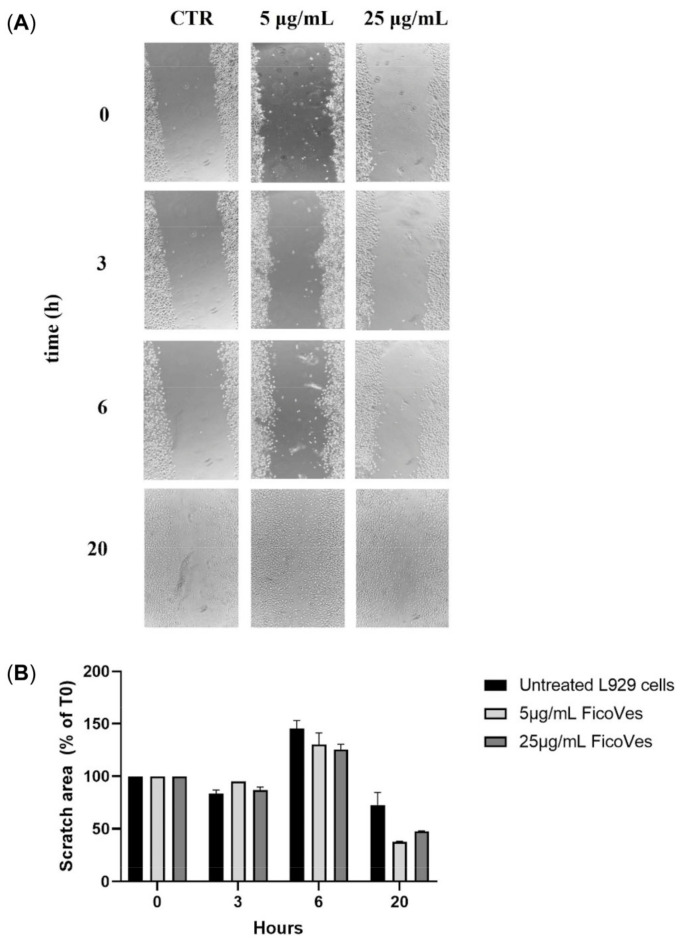
Wound closure on L929 cells monolayers treated with FicoVes. (**A**) Images of L929 monolayers at 0, 3 h , 6 h, and 20 h after the injury and the treatment with different concentrations (5 and 25 μg/mL) of FicoVes. (**B**) Histogram showing percentages of wound size at different time points calculated relative to the percentage of the wound area at T.0, which is considered 100%. The results are represented as mean ± SD of three experiments.

Given that one of the most common applications of vesicles in the biomedical field is epithelial repair [39], and considering the antioxidant and anti-inflammatory properties of FicoVes, we aimed to investigate whether FicoVes could contribute to epithelial repair and potentially have future applications in this area. To investigate this, we conducted a scratch test using L929 cell monolayers, which were mechanically scratched to simulate wound damage. We treated the L929 cell monolayers with different concentrations of FicoVes and took pictures at various time points: 3, 6, and 20 h post-treatment. To ensure that our results were not influenced by the proliferation rate of these cells, which have a doubling time of approximately 24 h , we conducted the experiment maximum at the 20 h treatment, using conditions that minimized the impact of cell proliferation on the results. As shown in Figure 7, at 6 h, treatment with FicoVes accelerated wound closure compared to the untreated control in a dose-dependent manner. This effect was more pronounced at 20 h, where nearly complete wound closure was observed in the treated cells compared to the control. Overall, these data illustrate the effect of FicoVes in the epithelial repair process on murine fibroblast monolayers.

## 4. Discussion

The results obtained in this study highlight the potential of Opuntia ficus-indica-derived vesicles (FicoVes) in exhibiting antioxidant, anti-inflammatory, and pro-healing properties, suggesting their promising application in biomedical fields.

Our nanoparticle tracking analysis (NTA) and dynamic light scattering (DLS) analyses confirmed that FicoVes possess a homogeneous size distribution, with an average of approximately 170 nm. This consistency between NTA, DLS, and scanning electron microscopy results (Figure 2) underscores the uniformity and stability of FicoVes in aqueous solution, with a zeta potential of −20.9 ± 6.34 mV, which is indicative of their colloidal stability.

The absorption spectra (240–700 nm) of FicoVes revealed a peak at 258 nm, corresponding to bioactive compounds such as flavonoids and phenolic acids. This is consistent with the known UV absorption profiles of phytocompounds [32], which are abundant in plant-derived vesicles. Moreover, the detection of a peak at 485 nm suggests the presence of carotenoids [33]. Fluorescence analysis confirmed the presence of these bioactive molecules within the vesicles, with a significant increase in fluorescence at 488 nm in FicoVes compared to plant cells, indicating an enrichment of these compounds. This suggests that FicoVes concentrate certain bioactive phytocomplexes, distinguishing them from animal-derived vesicles.

The antioxidant potential of FicoVes was evaluated through DPPH and FRAP assays, demonstrating a notable free radical scavenging capacity. Interestingly, the antioxidant activity of intact FicoVes was lower than that of sonicated vesicles, suggesting that the bioactive antioxidant compounds may be encapsulated within the vesicles and gradually released over time. This gradual release is consistent with the increasing antioxidant activity of intact FicoVes observed in the DPPH assay over time, as compared to the more immediate effect seen in sonicated vesicles. The hypothesis that the antioxidant components are not immediately available but become accessible as the vesicles release their contents over time may explain this pattern.

The FRAP assay further supported the antioxidant potential of FicoVes, as they exhibited sustained reducing properties for up to 6 h. These results highlight the robust antioxidant capabilities of FicoVes, with potential implications for their use in reducing oxidative stress.

Our results showed that FicoVes do not significantly affect the viability of several cell lines after 24 h of treatment, indicating their cytocompatibility. The cell viability test and hemolysis test confirmed the safety of FicoVes, supporting their potential for biomedical applications.

The anti-inflammatory potential of FicoVes was investigated by assessing their effects on cytokine expression in LPS-stimulated PBMCs. The data show that FicoVes reduced the expression of pro-inflammatory cytokines, such as IL1β and TNFα, in a dose-dependent manner. In addition, FicoVes increased the expression of anti-inflammatory cytokines IL10 and IL4, suggesting a dual modulatory role in inflammation. Importantly, the increase in IL10 expression (mRNA and protein) was observed both in LPS-treated and non-LPS-treated cells, confirming that FicoVes themselves are capable of upregulating anti-inflammatory responses (Figure S2). This supports the hypothesis that FicoVes contain bioactive compounds that can modulate the immune response independently of external inflammatory stimuli.

The reduction in pro-inflammatory cytokine expression, coupled with the increase in anti-inflammatory cytokines, highlights the potential of FicoVes to attenuate inflammatory responses. However, further studies are required to fully elucidate the underlying mechanisms and to confirm these findings in more complex models.

Given the antioxidant and anti-inflammatory properties of FicoVes, we investigated their role in the epithelial repair process. The scratch test on L929 fibroblasts revealed that FicoVes accelerated wound closure in a dose-dependent manner. This effect was most pronounced at 20 h, where nearly complete closure was observed. These results suggest that FicoVes could play an important role in promoting tissue repair, possibly due to their ability to reduce oxidative stress and modulate inflammation, both of which are critical processes in the epithelial repair process.

## 5. Conclusions

In this study, we propose, for the first time, the bio-fabrication of new nanovesicles from prickly pear fruit juice using a simple, efficient, cost-effective and reproducible production process. The FicoVes produced have an average diameter of 170 nm, negative zeta potential, and spheroidal morphology. In vitro, FicoVes show good hemocompatibility and cytocompatibility. Overall, our results suggest that FicoVes possess a unique combination of antioxidant, anti-inflammatory, and tissue repair-promoting properties. These properties, along with their biocompatibility and ability to modulate gene expression, make FicoVes a promising candidate in the field of nutrigenomics for future therapeutic applications, particularly in dermatology for the treatment of skin diseases such as psoriasis, dermatitis, and eczema. The use of gel or patch formulations containing these vesicles could be beneficial in preserving their chemical and physical characteristics. Furthermore, the use of patches could be useful for controlling the release of these vesicles over time during a topical application. Further studies, including in vivo models, are necessary to validate these findings and fully explore the therapeutic potential of FicoVes.

## Data Availability

Not applicable.

## Supplementary Materials

The following supporting information can be downloaded at: https://www.mdpi.com/article/10.3390/cells13211756/s1, Figure S1: Size distribution of FicoVes obtained through NTA analysis; Figure S2: Effect of FicoVes on expression of anti-inflammatory cytokines IL10 and IL4; Figure S3: Effect of FicoVes on protein expression of pro-inflammatory cytokines IL1β and anti-inflammatory cytokines IL10.

## Abbreviations

AKT	Protein kinase B (a cell-signaling protein)
ANOVA	Analysis of Variance
cDNA	Complementary DNA
DCFH-DA	2’:7’-Dichlorodihydrofluorescein Diacetate
DLS	Dynamic Light Scattering
DPPH	2:2-Diphenyl-1-picrylhydrazyl (used in antioxidant assays)
EVs	Extracellular Vesicles
FRAP	Ferric Reducing Antioxidant Power
IL10	Interleukin 10
IL1β	Interleukin 1 Beta
IL4	Interleukin 4
L929	A murine fibroblast cell line
LPS	Lipopolysaccharide
MTT	Thiazolyl Blue Tetrazolium Bromide
NTA	Nanoparticle Tracking Analysis
PBMCs	Peripheral Blood Mononuclear Cells
PBS	Phosphate Buffered Saline
PDNVs	Plant-Derived Nanovesicles
RAW 264.7	A murine macrophage cell line
RBCs	Red Blood Cells
ROS	Reactive Oxygen Species
RT-QPCR	Real-Time Quantitative Polymerase Chain Reaction
SD	Standard Deviation
SEM	Scanning Electron Microscopy
SH-SY5Y	A human neuroblastoma cell line
TBH	Tert-Butyl Hydroperoxide
TEAC	Trolox Equivalent Antioxidant Capacity
TNFα	Tumor Necrosis Factor Alpha
