# Supplementary material for: New Nanovesicles from Prickly Pear Fruit Juice: A Resource with Antioxidant, Anti-Inflammatory, and Nutrigenomic Properties
Source: Cells. 2024 Oct 23;13(21):1756. doi: 10.3390/cells13211756 (PMC11544800; doi:10.3390/cells13211756)
Supplement: Supplementary file 1 [file cells-13-01756-s001.zip › cells-3256997-supplementary.pdf]

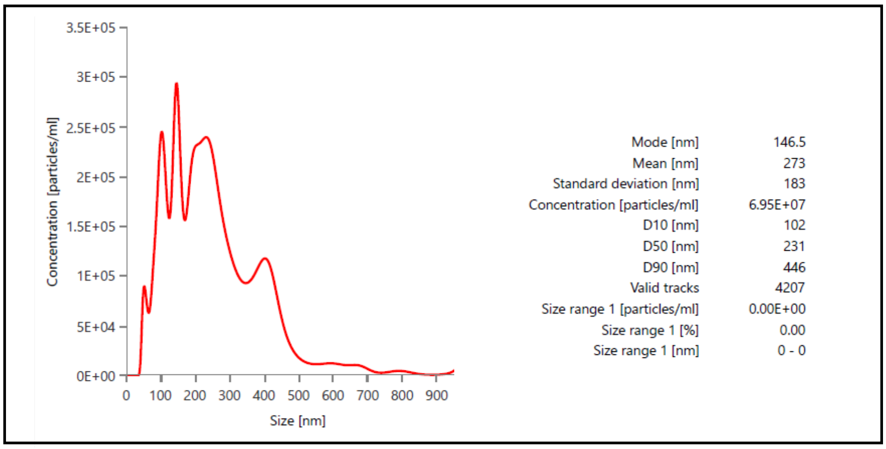

Figure S1. Size distribution of FicoVes obtained through NTA analysis. FicoVes at a concentration of 50 µg/mL were measured using the Bradford protein assay, a dye-binding method for determining total protein concentration.

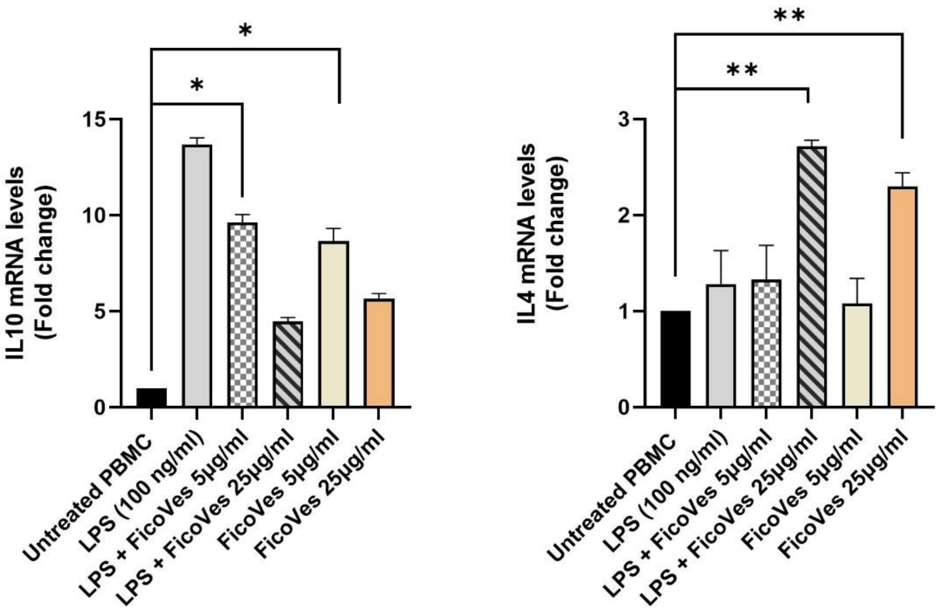

Figure S2. Effect of FicoVes on expression of anti-inflammatory cytokines IL10 and IL4. Data in the histograms are presented as the mean of three different experiments. Statistical significance was determined by comparison with the control group not treated FicoVes. Statistical analysis performed with one-way analysis of variance (ANOVA) and Tukey's multiple comparisons test. Differences were considered significant at * $p < 0.05$, *** $p < 0.005$.

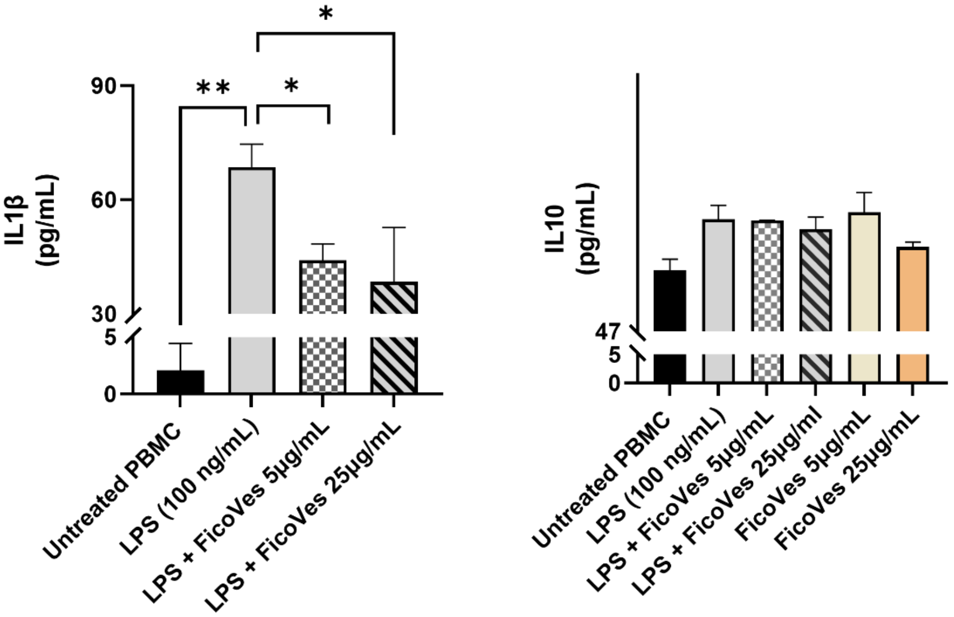

Figure S3. Effect of FicoVes on protein expression of pro-inflammatory cytokines IL1β and anti-inflammatory cytokines IL10 by ELISA test. Data in the histograms are presented as the mean of three different experiments. Statistical significance was determined by comparison with the control group not treated FicoVes. Statistical analysis performed with one-way analysis of variance (ANOVA) and Tukey's multiple comparisons test. Differences were considered significant at * $p < 0.05$, *** $p < 0.005$.
